# Beyond numbers: integrating qualitative analysis into quantitative sensory testing for neuropathic pain

**DOI:** 10.3389/fpain.2024.1351602

**Published:** 2024-02-28

**Authors:** Martine Bordeleau, Matthieu Vincenot, Miroslav Bačkonja, Yenisel Cruz-Almeida, Julia Forstenpointner, Lynn Gauthier, Serge Marchand, Catherine Mercier, Don Daniel Ocay, Michel PrudHomme, Hélène Ruel, Jan Vollert, Guillaume Léonard

**Affiliations:** ^1^ Research Center on Aging, CIUSSS de l’Estrie-CHUS, Sherbrooke, QC, Canada; ^2^ Faculty of Medicine and Health Sciences, Université de Sherbrooke, Sherbrooke, QC, Canada; ^3^ National Center for Complementary and Integrative Health, National Institutes of Health, Bethesda, MD, United States; ^4^ Pain Research & Intervention Center of Excellence, University of Florida Colleges of Dentistry & Medicine, Gainesville, FL, United States; ^5^ Department of Community Dentistry & Behavioral Science, University of Florida College of Dentistry, Gainesville, FL, United States; ^6^ Department of Neuroscience, University of Florida College of Medicine, Gainesville, FL, United States; ^7^ Division of Neurological Pain Research and Therapy, Department of Neurology, University Hospital Schleswig-Holstein, Kiel, Germany; ^8^ Department of Family and Emergency Medicine, Faculty of Medicine, Université Laval, Quebec City, QC, Canada; ^9^ Équipe de Recherche Michel-Sarrazin en Oncologie Psychosociale et Soins Palliatifs, Quebec City, QC, Canada; ^10^ Oncology Division, CHU de Québec-Université Laval Research Center, Quebec City, QC, Canada; ^11^ Université Laval Cancer Research Center, Quebec City, QC, Canada; ^12^ Département de chirurgie, Faculté de médecine et des sciences de la santé de l’université de Sherbrooke, Sherbrooke, QC, Canada; ^13^ Centre de Recherche du Centre Hospitalier de l’Université de Sherbrooke, Sherbrooke, QC, Canada; ^14^ Centre Interdisciplinaire de Recherche en Réadaptation et Intégration sociale, CIUSSS de la Capitale-Nationale, Quebec City, QC, Canada; ^15^ École des Sciences de la Réadaptation, Faculté de Médecine, Université Laval, Quebec City, QC, Canada; ^16^ Critical Care, and Pain Medicine, Department of Anesthesiology, Boston Children’s Hospital, Boston, MA, United States; ^17^ Department of Anaesthesia, Harvard Medical School, Boston, MA, United States; ^18^ Département de Chirurgie, Faculté de Médecine, Université Laval, Quebec City, QC, Canada; ^19^ Centre de Recherche du CHU-Université Laval, Quebec City, QC, Canada; ^20^ Department of Clinical Sciences, Faculty of Veterinary Medicine, Université de Montréal, Saint-Hyacinthe, QC, Canada; ^21^ Department of Clinical and Biomedical Sciences, Faculty of Health and Life Sciences, University of Exeter, Exeter, United Kingdom; ^22^ School of Rehabilitation, Faculty of Medicine and Health Sciences, Université de Sherbrooke, Sherbrooke, QC, Canada

**Keywords:** quantitative, qualitative, sensory testing, mixed approach, benefits, limitations

## Abstract

This article investigates the benefits of adopting qualitative and quantitative sensory testing (QQST) in sensory assessment, with a focus on understanding neuropathic pain. The innovative QQST method combines participant qualitative experiences with quantitative psychophysical measurements, offering a more varied interpretation of sensory abnormalities and normal sensory function. This article also explores the steps for the optimization of the method by identifying qualitative signs of sensory abnormalities and standardizing data collection. By leveraging the inherent subjectivity in the test design and participant responses, the QQST method contributes to a more holistic exploration of both normal and abnormal sensory experiences. This article positions the QQST approach as a foundational element within the Sensory Evaluation Network, uniting international experts to harmonize qualitative and quantitative sensory evaluation methods.

## 1 Sensory assessment breakthroughs

Neuropathic pain results from a lesion or disorder in the somatosensory nervous system (1), the management of which relies on in-depth sensory evaluation to understand its origins and manifestations, and guide treatment. This assessment can be facilitated by applying psychophysical methods, which evaluate the connection between a stimulus and the perceived sensation (2). This, in turn, facilitates precise diagnosis and differentiation of neuropathic pain from other types of pain (3).

In traditional clinical settings, sensory evaluation focuses on assessing the functionality of specific sensory receptors as they react to diverse stimuli, such as heat, cold, pressure or vibration (3, 4). Clinicians often rely on their judgment or compare the affected areas with unaffected ones to identify sensory irregularities. While these assessments can provide valuable information, they are often subjective, vary between clinicians, may overlook subtle sensory abnormalities, and offer no possibility to compare between limbs in the presence of widespread pain.

The precision of sensory evaluation has been significantly enhanced by advances in medical technology and techniques. Modern tools like quantitative sensory testing (QST) allow healthcare professionals to quantitatively assess sensory function. The goal of this non-invasive evaluation method is to quantify a participant's subjective response to a specific stimulus using a standardized and validated procedure (5). In this context, a sensory stimulus (e.g., thermal or mechanical) refers to the application of a validated thermal or mechanical testing device to the skin that is designed to elicit a specific sensory response from an individual. It may involve reaction time or not (6). The reaction time is the time it takes for sensory receptors in the stimulated skin area to be activated, nerve impulses to be transmitted to the brain, signals to be processed, and motor commands to be transmitted to end the test (6). During this time, the stimulus intensity is continuously increased (or decreased), influencing the detected threshold value, which is the point at which a sensory stimulus becomes perceptible or induces a response. Two common approaches are usually used to evaluate the response of the participant to the stimulus: (1) the method of limits, which involves a gradual change in stimulus intensity until the subject begins to feel its onset or disappearance and relies on the subject's reaction time (7); and (2) the method of levels, with stimulus intensity changing in steps and depend on the participant's response to the previous step (6). By quantifying sensory perceptions, statistical analyses can assess the impact of a treatment, for example, by comparing results obtained at various timepoints and during follow-up (5). QST has also been employed to categorize patients into subgroups of neuropathic pain based on their sensory patterns, allowing for phenotype-stratified trials and treatments (8–10). It is important to note that QST is a method focused only on assessing responses directly resulting from a stimulus. The method *does not* involve the measurement of sensations occurring seconds after the stimulus, even though some patients may report sensations like prickling or continued burning after completing a stimulus. Moreover, the method *does not* address the quality of ongoing pain, and there is a potential oversight of pain attacks unless they are provoked by one of the QST stimuli. It has been proposed that during the QST procedure, the exploration of participants' descriptions or interpretations of sensory perception could potentially improve the identification of sensory abnormalities (11, 12).

In previous research, the team led by Baad-Hansen investigated the agreement between QST and qualitative sensory assessments (13–16). They compared QST results collected using the DFNS protocol for extraoral application with qualitative sensory data from healthy individuals, patients suffering from orofacial pain, and individuals with damage to the trigeminal nerve. Qualitative sensory testing evaluated touch sensitivity with Q-tip stroke, cold using a cooled stainless-steel spatula, and pinprick with dental probe or toothpick (13–16). For each stimulus, patients were invited to indicate whether they perceived it as “more intense” (indicating hypersensitivity), “less intense” (indicating hyposensitivity), or “the same” (indicating normal sensitivity) on the affected side in comparison to the unaffected side. These studies collectively demonstrate a varied level of agreement between quantitative and qualitative sensory testing methods, with agreement percentages spanning a broad spectrum (47%–100%). In certain cases, quantitative tests did not detect abnormalities that participants could articulate using qualitative descriptions. This range in findings underscores the complexity of accurately measuring and interpreting sensory function and the importance of integrating both quantitative and qualitative approaches to capture a more comprehensive picture of sensory abnormalities.participantsparticipantsparticipants' experiences.

Our team recently proposed a standardized mixed-methods approach that combines participant experiences with quantitative measurements to create a comprehensive sensory assessment (12). We designated this innovative approach as qualitative and quantitative sensory testing (QQST). Despite this initiative, substantial work remains to refine this method. While the proposed QQST approach could provide valuable insights, it is not without limitations. For example, adding a qualitative component brings in a level of complexity that goes beyond a strictly protocol-driven and easily interpretable structure. The inclusion of qualitative elements may introduce variability and nuances that could pose challenges in terms of standardization and quick interpretation. Finding a balance between the richness of qualitative data and the need for efficiency in clinical assessments remains a challenge. Moreover, the interpretation of qualitative variables may be more subjective, potentially introducing a degree of variability in the analysis process. Therefore, careful thought and improvement of the methodology are necessary to capture the benefits of qualitative integration while dealing with these limitations. The purpose of this article is to investigate the benefits of adopting QQST and to explore the necessary steps to further its development and potential implementation.

## 2 Overcoming QST limitations with qualitative insights

Qualitative information can be collected during the QST procedure through open-ended and directed questions, encouraging patients to describe their sensory perceptions in their own words. This process captures the nuances of sensory perception, including the quality, intensity, and unique characteristics of sensations. The qualitative data collected can be systematically analyzed, categorized, and coded to create a classification system that complements quantitative data.

**Figure 1:**
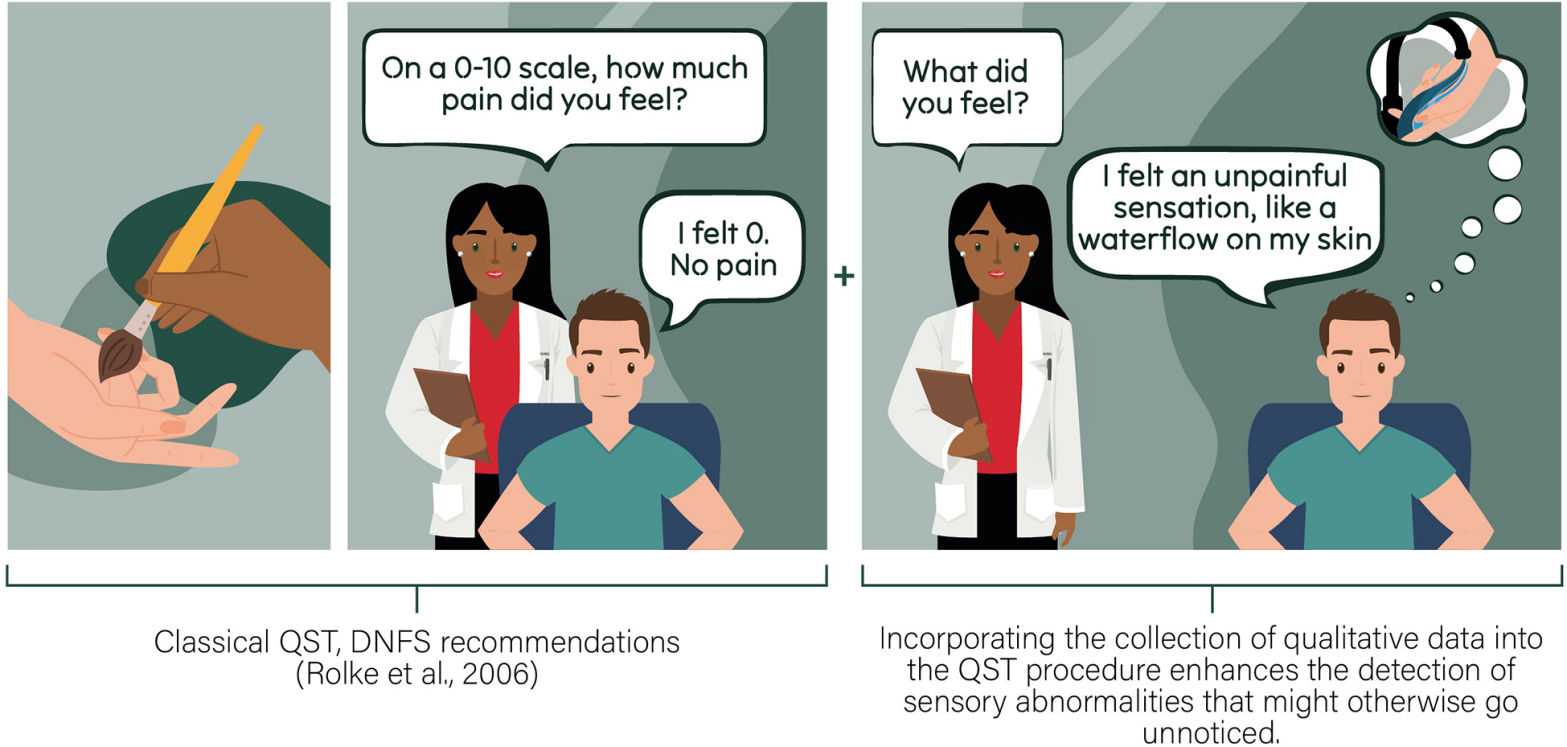
This scenario is based on a real-life event witnessed by our team during a previous QST assessment, in which the subject initially described a normal brush sensation on his skin in an unaffected area. However, in the painful area, the subject reported a very different sensation of water flowing on his skin (12). This situation illustrates how traditional QST methods can miss critical information that could indicate the presence of unusual sensations that could signal sensory abnormalities. Relying solely on traditional QST methods may result in an incomplete understanding of the sensory profile.

The integration of qualitative evaluation into QST brings several advantages. First, it facilitates an all-inclusive understanding of sensory abnormalities by combining objective measurements from QST with participants' unique descriptions, or interpretations of sensory perception, allowing for a comprehensive view that considers both clinical data and subjective experiences. Secondly, the integration recognizes and respects the individuality of sensory perception and pain, leading to a more personalized evaluation. This personalized approach could enhance the precision of diagnosis and treatment planning (17, 18). Additionally, the inclusion of qualitative evaluation improves sensitivity by capturing subtle sensory abnormalities that may be overlooked in a purely quantitative assessment (Figure 1). This combined methodology not only ensures a more thorough evaluation, but also provides a standardized procedure that can be replicated across different settings, promoting comparability and consistency in sensory assessments.

## 3 Call to action

The subsequent sections explain the essential actions required to advance the development of QQST, emphasizing the need for a clear classification system and ongoing refinement through expert collaboration. The work involves exploring qualitative signs, standardizing data collection, and optimizing the method though a collective effort.

### 3.1 A clear classification

The International Association for the Study of Pain (IASP) terminology was established in 1979 and updated over the years by various multinational, multidisciplinary Task Forces involving numerous researchers in the field (1). To facilitate effective integration of the qualitative aspect into QST, it is crucial to have a clear classification of terms associated with the presence of sensory abnormalities.

The available definitions significantly influenced our team's work in establishing a classification of sensory abnormalities detectable during the QQST procedures. During this reflective process, our team observed overlaps in some of the definitions proposed by the IASP. For instance, the term hyperesthesia encompasses both allodynia and hyperalgesia, while hypoesthesia includes hypoalgesia. Consequently, our team has introduced a consolidated classification with clear distinctions between each category to prevent overlap (Table 1). This approach aims to eliminate ambiguities and provides a structured codebook for analytical purposes during data interpretation. Implementing this precaution prevents researchers from unintentionally grouping different phenomena under the same label, ensuring consistency and repeatability.

**Table 1:** Comparison of the terminology developed by IASP and the one proposed by our team.

	Terminology developed by IASP for general purpose	Terminology proposed by our team for QQST assessment
Analgesia	Absence of pain in response to stimulation which would normally be painful	Same definition; our team suggest replacing this word by “adolor” (from a- “absence” + -dolor “pain”)*
Hypoalgesia	Diminished pain in response to a normally painful stimulus	Same
Hypoesthesia	Decreased sensitivity to stimulation, excluding the special senses	Decreased nonpainful sensitivity to an innocuous stimulation
Anesthesia	None	Absence of sensation in response to an innocuous stimulus; our team suggest replacing this word by “asensation” (from a- “absence” + -sensation)*
Hyperalgesia	Increased pain from a stimulus that normally provokes pain	Same
Hyperesthesia	Increased sensitivity to stimulation, excluding the special senses; hyperesthesia may refer to various modes of cutaneous sensibility including touch and thermal sensation without pain, as well as to pain	Increased nonpainful sensitivity to an innocuous stimulation
Allodynia	Pain due to a stimulus that does not normally provoke pain	Same
Paresthesia	An abnormal sensation, whether spontaneous or evoked; an abnormal sensation that is not unpleasant	Abnormal nonpainful sensation experienced by the subject, whether spontaneous or evoked during the QQST session
Dysesthesia	An unpleasant abnormal sensation, whether spontaneous or evoked; an abnormal sensation that is considered to be unpleasant	Abnormal painful sensation experienced by the subject, whether spontaneous or evoked during the QQST session

* Terms Like analgesia and anesthesia are commonly used to describe the administration of analgesic or anesthetic agents to patients undergoing surgeries and major medical procedures. Introducing new terms may help prevent confusion.

**Figure 2:**
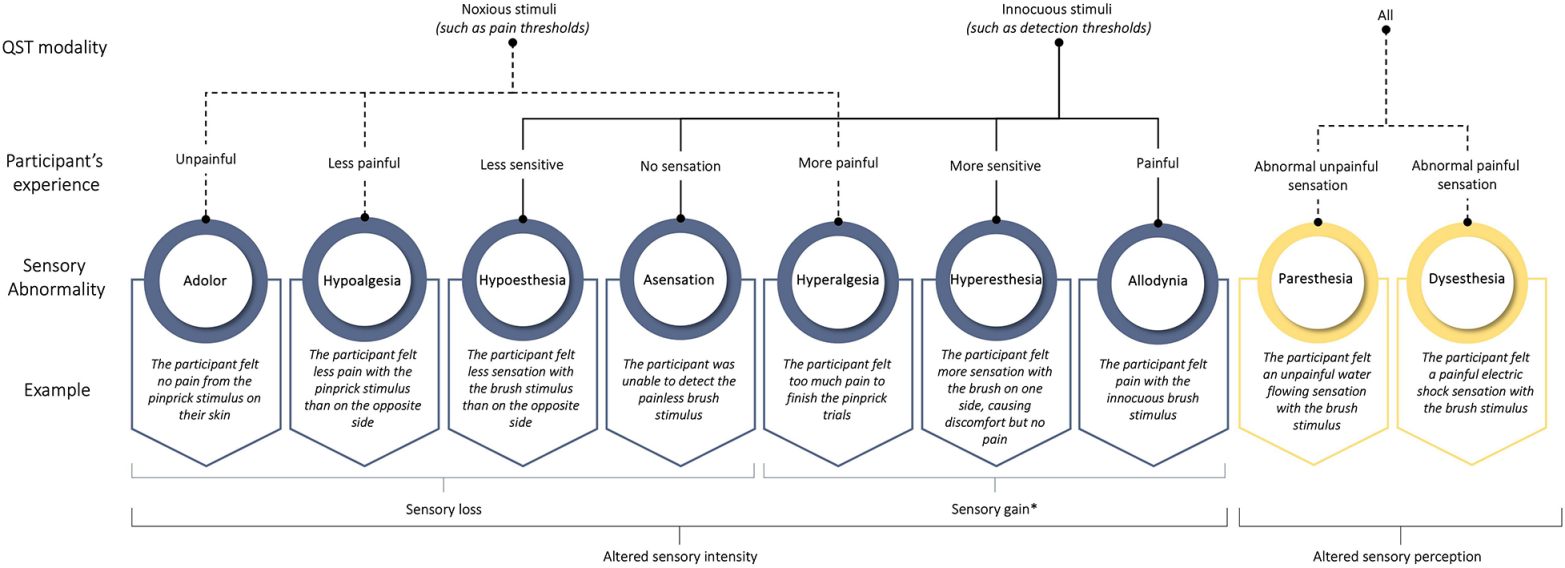
Potential classification of qualitative signs of sensory abnormalities observed during the QQST procedure. *When we use the term “sensory gain”, we refer to an increased sensitivity or enhanced perception of sensory stimuli, which is contrary to “sensory loss” that indicates reduced or absent sensation.

Our proposed classification (Figure 2) helps to standardize terminology by providing a deductive codebook to work with when analyzing data. Each qualitative observation in our classification could be assigned into two QST modality categories: (1) Noxious stimuli, which are specifically used to induce pain by activating nociceptors, involve tests like pain and tolerance thresholds; and (2) Innocuous stimuli, which aim to induce sensations that are not associated with pain, involve tests like detection thresholds and the detection of allodynia. Subsequently, based on subject experiences, collected qualitative observation could be categorized into two groups: (1) altered sensory intensity (i.e., an increase or decrease in the level of sensitivity experienced and reported by the patient) and/or (2) altered sensory perception (e.g., brush sensation felt like “electrical shock”). In the first group, altered sensory intensity could be divided into the following subgroups: analgesia, hypoalgesia, hypoesthesia, anesthesia, hyperalgesia, hyperesthesia, and allodynia. In the second group, altered sensory perception could be classified into the following subgroups: paresthesia and dysesthesia.

Still, this classification is far from optimal. For example, operational terms such as analgesia and anesthesia are frequently employed, suggesting the administration of analgesic or anesthetic agents to patients undergoing surgeries and significant medical procedures. It might be more prudent to establish new terms to avoid any confusion. For example, “adolor” (from a- “absence” + -dolor “pain”) may be used to denote a lack of reaction to painful stimuli, potentially serving as a substitute for the term analgesia. Similarly, “asensation” (from a- “absence” + -sensation) could be used to signify the absence of nonpainful sensations, providing a potential substitute for anesthesia in the context of QQST.

### 3.2 Identifying all qualitative signs of sensory abnormalities during QQST

Previously, our team categorized 630 qualitative observations of sensory abnormalities collected during a QST study with participants experiencing complex regional pain syndrome or failed back surgery syndrome (12). It is essential to expand this work to include other populations, as the significance of this work lies in its potential to offer enhanced guidance for assessors in classifying sensory abnormalities. For example, in our tests to find out when pain is elicited from a gradual/progressive increase of cold, heat, or pressure stimulations, we found that not everyone felt the intensity of the stimulus increase accordingly. At the start, which is supposed to be painless, some participants did not feel a gradual increase in sensation. Some participants observed that initially, there was no sensation as the intensity of the stimulus gradually increased. However, the sensation unexpectedly started to intensify, evolving into an unexpectedly powerful and painful experience (12). In these cases, the expected gradual increase in non-painful sensation, followed by the onset of pain sensation, did not occur as anticipated (12). According to our classification, we could interpret this phenomenon as a period of asensation during the innocuous phase of stimulus presentation when the sensation starts to increase, which could be followed by a period of hyperalgesia during the noxious phase. Another interesting example observed by our team (12) involves referred sensations where the perception of the tested stimulus occurs in a different part of the body than the one being tested (19). For example, a participant felt a non-painful sensation on their right hand's top while an innocuous stimulus was applied to the left hand; there was no reported sensation on the left hand (12). These examples underscore the complexity of the task and the necessity for a more in-depth exploration that could lead to modifications in the proposed classification.

### 3.3 Standardizing the data collection process and analysis

To be adequate, the data collection in QQST should strike a balance between the quantity of gathered data and the duration of the testing period. In both research and clinical settings, a traditional QST session may extend from 30 min to a few hours, influenced by factors such as the number of sensory modalities assessed, protocol complexity, and specific testing goals. It is important to assess the risks associated with prolonged data collection, particularly for a test relying on participants' subjective evaluation to identify thresholds. Adding a qualitative component to the QST approach may pose a limitation as it could increase the duration of the procedure. Based on our experience, the qualitative aspect can be mostly evaluated during the waiting periods of the traditional QST procedure. Furthermore, by verbally engaging participants during these intervals, we noted that it helps maintain their concentration levels throughout a procedure that can be tedious and potentially monotonous.

Our team is currently developing a preliminary draft of a standardized procedure inspired by QST procedures from the German Research Network on Neuropathic Pain (20) and Quebec Pain Research Network (21), aiming to integrate qualitative assessment during the usual waiting periods in the QST procedure. This procedure will be the subject of participative research-action projects characterized by a cyclical, iterative, and cooperative nature, allowing for its optimization over time.

Additionally, integrating triangulation into QQST can significantly enhance the validity of sensory assessments during subsequent data analysis. This approach uses both qualitative and quantitative methods to cross-verify findings. When comparing qualitative and quantitative results, convergence occurs if participants consistently describe certain sensations qualitatively, and quantitative data indicates abnormalities in corresponding sensory thresholds (based on reference data), reinforcing result reliability. Additionally, qualitative insights complement quantitative data, providing a deeper understanding of the subjective aspects of sensory experiences. For example, if quantitative data reveals heightened sensitivity to a stimulus, qualitative data may elucidate the emotional toll or functional impact of this heightened sensitivity. Another example could involve the comparison of QQST results to patient-reported outcomes (such as pain diaries or questionnaires) and clinical evaluations (such as nerve conduction studies or skin biopsies). This triangulated approach helps to ensure that the findings are not solely reliant on a single method, data source, or examiner.

### 3.4 Continuous optimization through collective effort

As for any methods in sensory evaluation, the QQST approach would immensely benefit from exchange with experts in the field. In this regard, our team has founded the Sensory Evaluation Network, whose mission is to bring together international specialists in sensory evaluation to discuss issues related to various qualitative and quantitative approaches, aiming to reach a consensus on potential improvements. This initiative seeks to address the current lack of communication and collaboration among experts. Discussions will take place in a private forum, access to which will be restricted to members of the expert group who must sign and commit to a confidentiality agreement. The outcomes of these discussions will be published. The second objective of the Sensory Evaluation Network is to offer a platform for educating and training researchers and students aiming to improve their expertise in sensory evaluation. This platform will be available freely as a web page and updated based on the consensus reached within the private forum.

## 5 Conclusion

Advancements in sensory assessment, especially regarding neuropathic pain, have been driven by psychophysical methods and technological progress. Our QQST method innovatively combines participant qualitative experiences with quantitative measures, offering a better understanding of sensory perception. Ongoing efforts focus on refining QQST, by identifying additional qualitative signs and standardizing data collection. Collaboration through the experts in the field is important to optimize benefits and overcome inherent limitations.
